# 3-*O*-Benzyl-6-*O*-benzoyl-1,2-*O*-isopropil­idene-5-*C*-nitro­methyl-*a*-d-glucofuran­ose

**DOI:** 10.1107/S1600536808043353

**Published:** 2009-01-17

**Authors:** Begoña Pampín, Laura Valencia, Juan C. Estévez, Ramón J. Estévez

**Affiliations:** a Departamento de Química Orgánica, Universidade de Santiago de Compostela, 15782 Santiago de Compostela, Spain; b Departamento de Química Inorgánica, Facultade de Química, Universidad de Vigo, 36310 Vigo, Pontevedra, Spain

## Abstract

The title compound, C_24_H_27_NO_9_, is one of the epimers of the Henry reaction of 3-*O*-benzyl-6-*O*-benzoyl-2-*O*-isopropyl­idene-*a*-d-glucofuran-5-one with nitro­methane. The conformation of the five membered rings is as expected from the precursor compound and the mol­ecule is folded with a dihedral angle of 51.4 (2)° between the aromatic rings. One O—H⋯O hydrogen bond and some intra­molecular and inter­molecular C—H⋯O inter­actions are observed in the structure.

## Related literature

For the preparation of 3-*O*-benzyl-6-*O*-benzoyl-1,2-isopro­pyl­idene-*a*-d-*xilo*-hexofuran-5-one, the precursor of the title compound, and for the Henry reaction of the title compound with nitro­methane, see: Yoshikawa *et al.* (1990 ▶). For background to nitro­sugars as precursors of a wide range of natural and synthetic products, see: Chakraborty *et al.* (2002 ▶); Gruner *et al.* (2002 ▶); Lillelund *et al.* (2002 ▶); Ogawa & Morikawa (2005 ▶).
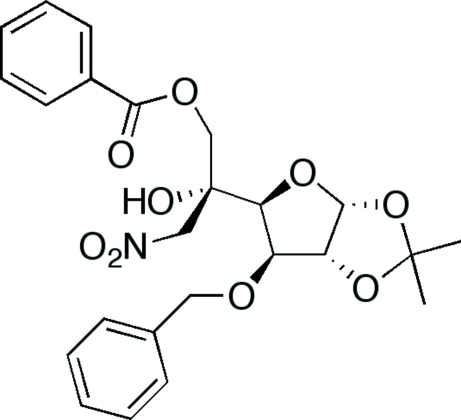


## Experimental

### 

#### Crystal data

C_24_H_27_NO_9_
*M* *_r_* = 473.47
Orthorhombic, 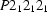

*a* = 9.5080 (12) Å
*b* = 11.8190 (16) Å
*c* = 21.395 (3) Å
*V* = 2404.3 (5) Å^3^
*Z* = 4
Mo *K*α radiation
μ = 0.10 mm^−1^
*T* = 113 (2) K
0.47 × 0.29 × 0.13 mm

#### Data collection

Bruker SMART CCD 1000 diffractometer
Absorption correction: multi-scan (*SADABS*; Sheldrick 1996 ▶) *T*_min_ = 0.626, *T*_max_ = 0.982
4735 measured reflections
2692 independent reflections
1940 reflections with *I* > 2σ(*I*)
*R*_int_ = 0.037

#### Refinement

*R*[*F* ^2^ > 2σ(*F* ^2^)] = 0.047
*wR*(*F* ^2^) = 0.131
*S* = 1.10
2692 reflections
313 parameters
1 restraint
H atoms treated by a mixture of independent and constrained refinement
Δρ_max_ = 0.25 e Å^−3^
Δρ_min_ = −0.30 e Å^−3^

### 

Data collection: *SMART* (Bruker, 1998 ▶); cell refinement: *SAINT* (Bruker, 1998 ▶); data reduction: *SAINT*; program(s) used to solve structure: *SIR97* (Altomare *et al.*, 1999 ▶); program(s) used to refine structure: *SHELXL97* (Sheldrick, 2008 ▶); molecular graphics: *ORTEP-3 for Windows* (Farrugia, 1997 ▶); software used to prepare material for publication: *WinGX* publication routines (Farrugia, 1999 ▶).

## Supplementary Material

Crystal structure: contains datablocks I, New_Global_Publ_Block. DOI: 10.1107/S1600536808043353/zl2144sup1.cif

Structure factors: contains datablocks I. DOI: 10.1107/S1600536808043353/zl2144Isup2.hkl

Additional supplementary materials:  crystallographic information; 3D view; checkCIF report

## Figures and Tables

**Table 1:** Hydrogen-bond geometry (Å, °)

*D*—H⋯*A*	*D*—H	H⋯*A*	*D*⋯*A*	*D*—H⋯*A*
O28—H28⋯O9	0.84 (2)	1.83 (3)	2.628 (4)	157 (5)
C4—H4⋯O31^i^	1.00	2.44	3.084 (5)	122
C5—H5⋯O28^ii^	1.00	2.45	3.189 (5)	130
C5—H5⋯O31^ii^	1.00	2.49	3.352 (5)	144
C18—H18*A*⋯O32	0.99	2.46	3.000 (6)	114
C24—H24⋯O1^iii^	0.95	2.59	3.445 (5)	151
C26—H26⋯O8^iv^	0.95	2.60	3.514 (5)	162
C29—H29*A*⋯O27^v^	0.99	2.54	3.334 (5)	137

Symmetry codes: (i) 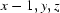; (ii) 
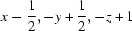
; (iii) 
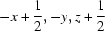
; (iv) 
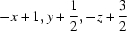
; (v) 
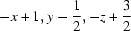
.

**Fig. 1.**
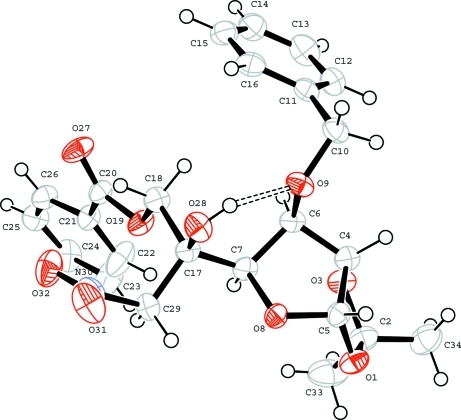
The molecular structure of the title compound (2), with atom labels and 50% probability displacement ellipsoids.

**Fig. 2.**
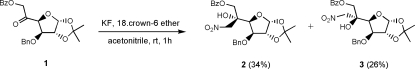
Chemical reaction scheme of the molecule (2).

## supplementary crystallographic information

### Comment

Nitrosugars are very important organic compounds because of their use as precursors of a wide range of natural and synthetic products with relevant properties (Gruner *et al.*, 2002) as aminopoliols (Lillelund *et al.*, 2002, Ogawa *et al.*, 2005), polihydroxilated amino acids (Chakraborty *et al.*, 2002), *etc*. The title nitrosugar compound 2 (C_24_H_27_NO_9_, Figure 1) is one of the epimers of the Henry reaction (Yoshikawa *et al.*, 1990) of 3-*O*-benzyl-6-*O*-benzoyl-2-*O*-isopropilidene-a-*D*-glucofuran-5-one (1) (Yoshikawa *et al.*, 1990) with nitromethane (See Figure 1). The molecular structure of the title compound is represented in Figure 2. Bond lengths and angles are within the expected values and confirm the bond orders giving in the Scheme. The compound crystallized in the orthorhombic space group *P*2_1_2_1_2_1_ with only one molecule in the asymmetric unit. The molecule is folded with a dihedral angle between the aromatic rings of 51.4 (2)°. The conformation of the five membered rings is as expected from the precursor compound (1). Some intramolecular and intermolecular H bond interactions have been observed in the structure. The intramolecular O28—H28···O9 H bond interaction shows a distance H28···O9 of 2.628 (4) Å and an angle of 157 (5)°. No π—π-stacking interactions have been observed in the structure of the title compound.

### Experimental

3-*O*-Benzyl-6-*O*-benzoyl-1,2-*O*-isopropilidene-5-*C*-nitromethyl-a-*D*-glucofuranose (2) and 3-*O*-benzyl-6-*O*-benzoyl-1,2-*O*-isopropilidene-5-*C*-nitromethyl-b-*L*-Idofuranose (3).

KF.2H_2_O (0.46 g, 4.90 mmol) and 18-crown-6 ether (0.82 g, 3.10 mmol) were added to a solution of 3-*O*-benzyl-6-*O*-benzoyl-1,2-isopropylidene-a-*D*-*xilo*-hexofuran-5-one (1) (1.19 g, 2.90 mmol) in acetonitrile (18 ml) and the resulting suspension was stirred at room temperature for 1 h. The reaction mixture was poured into ice water (50 ml) and extracted with ethyl acetate (3 m × 80 ml). The organic layers were then dried with anhydrous sodium sulfate, filtered and evaporated to give a residue wich was purificated by flash column chromatography (ethyl acetate/hexane 1:3) to give 3-*O*-benzyl-6-*O*-benzoyl-1,2-*O*-isopropilidene-5-*C*-nitromethyl-a-*D*-glucofuranose (2) (0.47 g, 34%) and 3-*O*-benzyl-6-*O*-benzoyl-1,2-*O*-isopropilidene-5-*C*-nitromethyl-b-*L*-Idofuranose (3) (0.36 g, 26%) as white solids that were crystallized from a mixture of ethylacetate and hexane.

3-*O*-Benzyl-6-*O*-benzoyl-1,2-*O*-isopropilidene-5-*C*-nitromethyl-a-*D*-glucofuranose (2): mp: 375–379 K. [*a*]_D_^22 ^-75.6° (c 1.00, CHCl_3_). IR (NaCl, cm^-1^): 3454 (OH); 2854–3064 (C_Ar_H); 1722 (CO); 1554, 1375 (NO_2_). ^1^H NMR (250 MHz, CDCl_3_) δ 1.32 (s, 3H, CH_3_); 1.46 (s, 3H, CH_3_); 4.30 (d, 1H, *J*_3,4_= 3.35 Hz, H-3); 4.38 (d, 1H, *J*_4,3_= 3.35 Hz, H-4); 4.42–4.47 (m, 2H, CH_2_NO_2_); 4.54–4.60 (m, 2H, H-6 + H-6'); 4.66 (d, 1H, *J*_2,1_= 3.65 Hz, H-2); 4.73 (d, 1H, *J*= 11.87 Hz, CHPh); 4.80 (s, 1H, OH); 4.91 (d, 1H, *J* = 11.87 Hz, CHPh); 6.02 (d, 1H, *J*_1,2_ = 3.65 Hz, H-1); 7.31–7.61 (m, 8H, 8 × HPh); 7.98–8.01 (m, 2H, 2 × HPh). ^13^CNMR (62.8 MHz, CDCl_3_) δ 26.17 (CH_3_); 26.56 (CH_3_); 65.34 (CH_2_); 72.40 (CH_2_); 73.05 (CH_2_); 77.62 (CH); 77.76 (C); 81.38 (CH); 82.99 (CH); 104.53 (CH); 112.19 (C); 128.21 (2 × C_Ar_H); 128.45 (2 × C_Ar_H); 128.67(C_Ar_H); 128.79 (2 × Ar C_Ar_H); 129.06 (C_Ar_); 129.54 (2 × C_Ar_H); 133.39 (C_Ar_H); 135.47 (C_Ar_); 165.64 (CO). MS (CI) *m*/*z* 474 [(*M*+H)^+^, 1]; 105 (38); 91 [(PhCH_2_)^+^, 81], 28 (100).

3-*O*-Benzyl-6-*O*-benzoyl-1,2-*O*-isopropilidene-5-*C*-nitromethyl-b-*L*-Idofuranose (3): mp: 382–384 K. [*a*]_D_^22 ^-38.0° (c 1.90, CHCl_3_). IR (NaCl, cm^-1^): 3454 (OH); 2854–3089 (C_Ar_H); 1722 (CO); 1554, 1375 (NO_2_). ^1^H NMR (250 MHz, CDCl_3_) δ 1.33 (s, 3H, CH_3_); 1.45 (s, 3H, CH_3_); 4.27 (d, 1H, *J*_3,4_ = 3.35 Hz, H-3); 4.31 (s, 1H, OH); 4.39 (1*H*, d, *J*_4,3_= 3.35 Hz, H-4); 4.49–4.64 (m, 5H, H-6 + H-6' + CH_2_NO_2_ + CHPh); 4.69 (d, 1H, *J*_2,1_ =3.35 Hz, H-2); 4.74 (d, 1H, *J* = 11.8 Hz, CHPh); 6.01 (d, 1H, *J*_1,2_= 3.35 Hz, H-1); 7.33–7.60 (m, 8H, 8 × H—Ph); 7.96–8.01 (m, 2H, 2 × H—Ph).^13^CNMR (62.8 MHz, CDCl_3_) δ 26.60 (CH_3_); 27.09 (CH_3_); 65.90 (CH_2_); 72.52 (CH_2_); 73.90 (C); 78.96 (CH_2_); 79.20 (CH); 81.80 (CH); 82.70 (CH); 104.90 (CH); 112.70 (C); 128.90 (4 × C_Ar_H); 129.20 (2 × C_Ar_H); 129.30 (2 × C_Ar_H); 129.80 (C_Ar_); 130.02 (C_Ar_H); 133.70 (C_Ar_H); 136.02 (C_Ar_); 166.05 (CO). MS (CI) *m*/*z*105 (38); 91 [(PhCH_2_)^+^, 86], 61 (100); 28 (87).

### Refinement

As the data were collected with Mo-Kα radiation and no heavy atoms present anomalous dispersion data are not reliable and Friedel opposites were thus merged before refinement. The hydrogen atom of the alcohol group, H28, was located in a difference density Fourier map and was refined isotropically. All other hydrogen atoms were located in calculated positions and were refined using a riding model with C-H diatnces of 0.95 to 1.0 Å and U_iso_(H) = U_eq_(C) of the adjacent carbon atom.

### Crystal data

**Table:**

C_24_H_27_NO_9_	*D*_x_ = 1.308 Mg m^−^^3^
*M**_r_* = 473.47	Mo *K*α radiation, λ = 0.71069 Å
Orthorhombic, *P*2_1_2_1_2_1_	Cell parameters from 915 reflections
*a* = 9.5080 (12) Å	θ = 2.6–24.4°
*b* = 11.8190 (16) Å	µ = 0.10 mm^−^^1^
*c* = 21.395 (3) Å	*T* = 113 K
*V* = 2404.3 (5) Å^3^	Prism, colourless
*Z* = 4	0.47 × 0.29 × 0.13 mm
*F*(000) = 1000	

### Data collection

**Table:**

Bruker SMART CCD 1000 diffractometer	2692 independent reflections
Radiation source: fine-focus sealed tube	1940 reflections with *I* > 2σ(*I*)
graphite	*R*_int_ = 0.037
ω scans	θ_max_ = 26.0°, θ_min_ = 1.9°
Absorption correction: multi-scan (*SADABS*; Sheldrick 1996)	*h* = −11→11
*T*_min_ = 0.626, *T*_max_ = 0.982	*k* = 0→14
4735 measured reflections	*l* = 0→26

### Refinement

**Table:**

Refinement on *F*^2^	Primary atom site location: structure-invariant direct methods
Least-squares matrix: full	Secondary atom site location: difference Fourier map
*R*[*F*^2^ > 2σ(*F*^2^)] = 0.047	Hydrogen site location: inferred from neighbouring sites
*wR*(*F*^2^) = 0.131	H atoms treated by a mixture of independent and constrained refinement
*S* = 1.10	*w* = 1/[σ^2^(*F*_o_^2^) + (0.0583*P*)^2^ + 1.021*P*] where *P* = (*F*_o_^2^ + 2*F*_c_^2^)/3
2692 reflections	(Δ/σ)_max_ < 0.001
313 parameters	Δρ_max_ = 0.25 e Å^−^^3^
1 restraint	Δρ_min_ = −0.30 e Å^−^^3^

### Special details

**Table:**

Geometry. All e.s.d.'s (except the e.s.d. in the dihedral angle between two l.s. planes) are estimated using the full covariance matrix. The cell e.s.d.'s are taken into account individually in the estimation of e.s.d.'s in distances, angles and torsion angles; correlations between e.s.d.'s in cell parameters are only used when they are defined by crystal symmetry. An approximate (isotropic) treatment of cell e.s.d.'s is used for estimating e.s.d.'s involving l.s. planes.
Refinement. Refinement of *F*^2^ against ALL reflections. The weighted *R*-factor *wR* and goodness of fit *S* are based on *F*^2^, conventional *R*-factors *R* are based on *F*, with *F* set to zero for negative *F*^2^. The threshold expression of *F*^2^ > σ(*F*^2^) is used only for calculating *R*-factors(gt) *etc*. and is not relevant to the choice of reflections for refinement. *R*-factors based on *F*^2^ are statistically about twice as large as those based on *F*, and *R*- factors based on ALL data will be even larger.

### Fractional atomic coordinates and isotropic or equivalent isotropic displacement parameters (Å^2^)

**Table:**

	*x*	*y*	*z*	*U*_iso_*/*U*_eq_	
O1	0.1700 (3)	0.0030 (2)	0.56353 (12)	0.0391 (7)	
C2	0.0591 (5)	0.0120 (3)	0.61034 (19)	0.0402 (11)	
O3	0.0623 (3)	0.1268 (2)	0.62962 (11)	0.0348 (7)	
C4	0.1148 (5)	0.1931 (3)	0.57862 (16)	0.0302 (9)	
H4	0.0397	0.2228	0.5503	0.036*	
C5	0.2170 (4)	0.1109 (3)	0.54653 (16)	0.0303 (9)	
H5	0.2143	0.1206	0.5001	0.036*	
C6	0.2093 (4)	0.2840 (3)	0.60556 (16)	0.0288 (9)	
H6	0.1677	0.3197	0.6437	0.035*	
C7	0.3420 (5)	0.2167 (3)	0.62059 (15)	0.0283 (9)	
H7	0.3265	0.1746	0.6606	0.034*	
O8	0.3531 (3)	0.1354 (2)	0.57021 (11)	0.0308 (6)	
O9	0.2419 (3)	0.3664 (2)	0.55803 (10)	0.0315 (6)	
C10	0.1342 (5)	0.4495 (3)	0.54682 (17)	0.0337 (10)	
H10A	0.0461	0.4106	0.5347	0.040*	
H10B	0.1632	0.4986	0.5116	0.040*	
C11	0.1070 (5)	0.5218 (3)	0.60372 (16)	0.0308 (9)	
C12	−0.0205 (5)	0.5153 (4)	0.63417 (18)	0.0382 (10)	
H12	−0.0940	0.4699	0.6177	0.046*	
C13	−0.0408 (6)	0.5759 (4)	0.6894 (2)	0.0477 (12)	
H13	−0.1286	0.5716	0.7104	0.057*	
C14	0.0640 (6)	0.6412 (4)	0.7134 (2)	0.0443 (11)	
H14	0.0490	0.6816	0.7512	0.053*	
C15	0.1910 (5)	0.6489 (3)	0.6834 (2)	0.0422 (11)	
H15	0.2640	0.6942	0.7005	0.051*	
C16	0.2130 (5)	0.5899 (3)	0.62766 (18)	0.0363 (10)	
H16	0.3001	0.5965	0.6062	0.044*	
C17	0.4799 (5)	0.2818 (3)	0.62561 (15)	0.0279 (9)	
C18	0.4703 (5)	0.3727 (3)	0.67693 (15)	0.0318 (9)	
H18A	0.5578	0.4182	0.6778	0.038*	
H18B	0.3903	0.4240	0.6684	0.038*	
O19	0.4505 (3)	0.3165 (2)	0.73631 (10)	0.0330 (7)	
C20	0.4824 (5)	0.3802 (3)	0.78726 (16)	0.0307 (9)	
C21	0.4595 (5)	0.3169 (3)	0.84646 (16)	0.0317 (9)	
C22	0.3918 (6)	0.2137 (4)	0.8481 (2)	0.0571 (15)	
H22	0.3611	0.1799	0.8102	0.069*	
C23	0.3680 (7)	0.1590 (5)	0.9043 (2)	0.0663 (17)	
H23	0.3222	0.0876	0.9051	0.080*	
C24	0.4120 (6)	0.2096 (4)	0.95958 (18)	0.0497 (13)	
H24	0.3915	0.1748	0.9985	0.060*	
C25	0.4849 (5)	0.3097 (4)	0.95812 (17)	0.0416 (11)	
H25	0.5193	0.3417	0.9959	0.050*	
C26	0.5087 (5)	0.3643 (3)	0.90188 (16)	0.0369 (10)	
H26	0.5584	0.4341	0.9011	0.044*	
O27	0.5252 (4)	0.4754 (2)	0.78355 (11)	0.0436 (8)	
O28	0.5152 (3)	0.3409 (2)	0.56949 (11)	0.0339 (7)	
C29	0.5974 (4)	0.1972 (3)	0.63705 (18)	0.0324 (9)	
H29A	0.5829	0.1593	0.6778	0.039*	
H29B	0.5963	0.1386	0.6040	0.039*	
N30	0.7345 (4)	0.2559 (3)	0.63692 (16)	0.0411 (9)	
O31	0.8021 (3)	0.2601 (3)	0.58777 (14)	0.0529 (9)	
O32	0.7732 (4)	0.3030 (3)	0.68513 (14)	0.0516 (9)	
C33	0.0979 (8)	−0.0629 (4)	0.6645 (2)	0.0663 (17)	
H33A	0.1894	−0.0396	0.6812	0.099*	
H33B	0.1033	−0.1417	0.6504	0.099*	
H33C	0.0264	−0.0563	0.6973	0.099*	
C34	−0.0801 (5)	−0.0168 (4)	0.5813 (2)	0.0530 (13)	
H34A	−0.1544	−0.0109	0.6129	0.080*	
H34B	−0.0771	−0.0942	0.5649	0.080*	
H34C	−0.0998	0.0360	0.5470	0.080*	
H28	0.434 (3)	0.366 (4)	0.561 (2)	0.060 (17)*	

### Atomic displacement parameters (Å^2^)

**Table:**

	*U*^11^	*U*^22^	*U*^33^	*U*^12^	*U*^13^	*U*^23^
O1	0.046 (2)	0.0339 (14)	0.0374 (14)	−0.0003 (14)	0.0047 (14)	−0.0101 (12)
C2	0.048 (3)	0.034 (2)	0.039 (2)	−0.002 (2)	0.008 (2)	−0.0016 (18)
O3	0.0463 (19)	0.0312 (13)	0.0269 (12)	0.0023 (14)	0.0063 (13)	0.0019 (11)
C4	0.038 (3)	0.0319 (18)	0.0208 (17)	0.0002 (18)	0.0030 (17)	0.0013 (15)
C5	0.034 (3)	0.033 (2)	0.0232 (17)	−0.0006 (18)	−0.0012 (17)	−0.0028 (15)
C6	0.040 (3)	0.0282 (18)	0.0180 (16)	0.0042 (18)	0.0027 (16)	0.0009 (14)
C7	0.039 (3)	0.0308 (19)	0.0148 (16)	0.0034 (18)	0.0016 (16)	−0.0045 (13)
O8	0.0351 (18)	0.0345 (13)	0.0228 (12)	0.0046 (13)	−0.0043 (11)	−0.0092 (10)
O9	0.0398 (18)	0.0338 (13)	0.0208 (11)	0.0091 (13)	0.0017 (12)	0.0046 (10)
C10	0.039 (3)	0.036 (2)	0.0263 (18)	0.0090 (19)	−0.0026 (18)	0.0021 (15)
C11	0.035 (3)	0.0298 (19)	0.0282 (18)	0.0045 (18)	−0.0009 (18)	0.0070 (15)
C12	0.033 (3)	0.045 (2)	0.036 (2)	0.003 (2)	0.0000 (19)	0.0016 (18)
C13	0.040 (3)	0.062 (3)	0.040 (2)	0.011 (3)	0.011 (2)	0.001 (2)
C14	0.051 (3)	0.046 (2)	0.036 (2)	0.009 (2)	0.000 (2)	−0.0074 (19)
C15	0.054 (3)	0.031 (2)	0.041 (2)	0.003 (2)	−0.006 (2)	−0.0039 (18)
C16	0.041 (3)	0.0318 (19)	0.036 (2)	0.0028 (19)	0.0047 (19)	0.0073 (17)
C17	0.040 (3)	0.0274 (18)	0.0167 (15)	0.0017 (18)	−0.0014 (16)	0.0017 (13)
C18	0.043 (3)	0.034 (2)	0.0186 (16)	0.000 (2)	−0.0009 (17)	0.0042 (14)
O19	0.052 (2)	0.0317 (13)	0.0157 (11)	−0.0066 (14)	−0.0016 (12)	−0.0007 (10)
C20	0.038 (3)	0.032 (2)	0.0218 (16)	−0.0028 (19)	−0.0013 (17)	−0.0042 (14)
C21	0.038 (3)	0.037 (2)	0.0201 (16)	−0.0037 (19)	0.0004 (17)	−0.0006 (15)
C22	0.085 (4)	0.060 (3)	0.027 (2)	−0.034 (3)	−0.009 (2)	0.0057 (19)
C23	0.087 (5)	0.071 (3)	0.040 (2)	−0.043 (3)	−0.010 (3)	0.019 (2)
C24	0.057 (3)	0.068 (3)	0.024 (2)	−0.007 (3)	0.001 (2)	0.014 (2)
C25	0.057 (3)	0.048 (2)	0.0192 (17)	0.006 (2)	−0.0037 (19)	−0.0037 (16)
C26	0.051 (3)	0.0364 (19)	0.0231 (17)	0.000 (2)	−0.0005 (18)	−0.0038 (15)
O27	0.079 (2)	0.0289 (14)	0.0231 (12)	−0.0101 (16)	−0.0052 (14)	−0.0029 (10)
O28	0.040 (2)	0.0450 (16)	0.0169 (11)	0.0035 (14)	0.0009 (12)	0.0047 (11)
C29	0.029 (3)	0.038 (2)	0.0301 (19)	0.0012 (19)	−0.0031 (18)	0.0020 (16)
N30	0.042 (2)	0.047 (2)	0.0340 (18)	0.0058 (19)	−0.0066 (17)	0.0115 (16)
O31	0.039 (2)	0.079 (2)	0.0404 (17)	0.0084 (18)	0.0052 (15)	0.0129 (16)
O32	0.056 (2)	0.061 (2)	0.0387 (16)	−0.0149 (18)	−0.0148 (16)	0.0018 (15)
C33	0.107 (5)	0.042 (2)	0.050 (3)	0.010 (3)	0.009 (3)	0.011 (2)
C34	0.048 (3)	0.049 (3)	0.062 (3)	−0.011 (2)	0.009 (3)	−0.009 (2)

### Geometric parameters (Å, °)

**Table:**

O1—C5	1.400 (5)	C17—O28	1.429 (4)
O1—C2	1.458 (5)	C17—C29	1.519 (5)
C2—O3	1.419 (5)	C17—C18	1.539 (5)
C2—C34	1.502 (7)	C18—O19	1.446 (4)
C2—C33	1.505 (6)	C18—H18A	0.9900
O3—C4	1.433 (4)	C18—H18B	0.9900
C4—C6	1.515 (5)	O19—C20	1.359 (4)
C4—C5	1.536 (5)	C20—O27	1.199 (5)
C4—H4	1.0000	C20—C21	1.487 (5)
C5—O8	1.419 (5)	C21—C22	1.379 (6)
C5—H5	1.0000	C21—C26	1.393 (5)
C6—O9	1.442 (4)	C22—C23	1.385 (6)
C6—C7	1.526 (6)	C22—H22	0.9500
C6—H6	1.0000	C23—C24	1.391 (6)
C7—O8	1.448 (4)	C23—H23	0.9500
C7—C17	1.524 (6)	C24—C25	1.371 (6)
C7—H7	1.0000	C24—H24	0.9500
O9—C10	1.439 (5)	C25—C26	1.384 (5)
C10—C11	1.510 (5)	C25—H25	0.9500
C10—H10A	0.9900	C26—H26	0.9500
C10—H10B	0.9900	O28—H28	0.84 (2)
C11—C12	1.378 (6)	C29—N30	1.476 (6)
C11—C16	1.388 (6)	C29—H29A	0.9900
C12—C13	1.394 (6)	C29—H29B	0.9900
C12—H12	0.9500	N30—O32	1.228 (4)
C13—C14	1.361 (7)	N30—O31	1.233 (5)
C13—H13	0.9500	C33—H33A	0.9800
C14—C15	1.370 (7)	C33—H33B	0.9800
C14—H14	0.9500	C33—H33C	0.9800
C15—C16	1.398 (6)	C34—H34A	0.9800
C15—H15	0.9500	C34—H34B	0.9800
C16—H16	0.9500	C34—H34C	0.9800
			
C5—O1—C2	110.1 (3)	C15—C16—H16	120.1
O3—C2—O1	104.7 (3)	O28—C17—C29	106.5 (3)
O3—C2—C34	110.9 (4)	O28—C17—C7	112.9 (3)
O1—C2—C34	109.7 (3)	C29—C17—C7	108.2 (3)
O3—C2—C33	109.5 (4)	O28—C17—C18	105.8 (3)
O1—C2—C33	108.0 (4)	C29—C17—C18	112.8 (3)
C34—C2—C33	113.7 (4)	C7—C17—C18	110.6 (3)
C2—O3—C4	108.0 (3)	O19—C18—C17	108.3 (3)
O3—C4—C6	107.7 (3)	O19—C18—H18A	110.0
O3—C4—C5	102.4 (3)	C17—C18—H18A	110.0
C6—C4—C5	104.1 (3)	O19—C18—H18B	110.0
O3—C4—H4	113.8	C17—C18—H18B	110.0
C6—C4—H4	113.8	H18A—C18—H18B	108.4
C5—C4—H4	113.8	C20—O19—C18	114.9 (3)
O1—C5—O8	112.6 (3)	O27—C20—O19	122.9 (3)
O1—C5—C4	104.9 (3)	O27—C20—C21	125.3 (3)
O8—C5—C4	106.8 (3)	O19—C20—C21	111.8 (3)
O1—C5—H5	110.8	C22—C21—C26	119.5 (3)
O8—C5—H5	110.8	C22—C21—C20	122.3 (3)
C4—C5—H5	110.8	C26—C21—C20	118.2 (3)
O9—C6—C4	109.8 (3)	C21—C22—C23	120.8 (4)
O9—C6—C7	108.8 (3)	C21—C22—H22	119.6
C4—C6—C7	101.6 (3)	C23—C22—H22	119.6
O9—C6—H6	112.0	C22—C23—C24	119.2 (4)
C4—C6—H6	112.0	C22—C23—H23	120.4
C7—C6—H6	112.0	C24—C23—H23	120.4
O8—C7—C17	109.0 (3)	C25—C24—C23	120.3 (4)
O8—C7—C6	104.4 (3)	C25—C24—H24	119.9
C17—C7—C6	117.6 (3)	C23—C24—H24	119.9
O8—C7—H7	108.5	C24—C25—C26	120.3 (4)
C17—C7—H7	108.5	C24—C25—H25	119.8
C6—C7—H7	108.5	C26—C25—H25	119.8
C5—O8—C7	109.6 (3)	C25—C26—C21	119.8 (4)
C10—O9—C6	115.2 (3)	C25—C26—H26	120.1
O9—C10—C11	112.0 (3)	C21—C26—H26	120.1
O9—C10—H10A	109.2	C17—O28—H28	97 (4)
C11—C10—H10A	109.2	N30—C29—C17	109.9 (3)
O9—C10—H10B	109.2	N30—C29—H29A	109.7
C11—C10—H10B	109.2	C17—C29—H29A	109.7
H10A—C10—H10B	107.9	N30—C29—H29B	109.7
C12—C11—C16	119.8 (4)	C17—C29—H29B	109.7
C12—C11—C10	120.0 (4)	H29A—C29—H29B	108.2
C16—C11—C10	120.1 (4)	O32—N30—O31	122.8 (4)
C11—C12—C13	119.6 (4)	O32—N30—C29	118.4 (4)
C11—C12—H12	120.2	O31—N30—C29	118.7 (3)
C13—C12—H12	120.2	C2—C33—H33A	109.5
C14—C13—C12	120.7 (5)	C2—C33—H33B	109.5
C14—C13—H13	119.7	H33A—C33—H33B	109.5
C12—C13—H13	119.7	C2—C33—H33C	109.5
C13—C14—C15	120.4 (4)	H33A—C33—H33C	109.5
C13—C14—H14	119.8	H33B—C33—H33C	109.5
C15—C14—H14	119.8	C2—C34—H34A	109.5
C14—C15—C16	119.9 (4)	C2—C34—H34B	109.5
C14—C15—H15	120.1	H34A—C34—H34B	109.5
C16—C15—H15	120.1	C2—C34—H34C	109.5
C11—C16—C15	119.7 (4)	H34A—C34—H34C	109.5
C11—C16—H16	120.1	H34B—C34—H34C	109.5
			
C5—O1—C2—O3	−11.5 (4)	C12—C13—C14—C15	0.5 (7)
C5—O1—C2—C34	107.5 (4)	C13—C14—C15—C16	0.3 (7)
C5—O1—C2—C33	−128.2 (4)	C12—C11—C16—C15	1.9 (6)
O1—C2—O3—C4	27.8 (4)	C10—C11—C16—C15	−174.4 (3)
C34—C2—O3—C4	−90.4 (4)	C14—C15—C16—C11	−1.5 (6)
C33—C2—O3—C4	143.4 (4)	O8—C7—C17—O28	59.3 (4)
C2—O3—C4—C6	−141.4 (4)	C6—C7—C17—O28	−59.2 (4)
C2—O3—C4—C5	−32.0 (4)	O8—C7—C17—C29	−58.3 (4)
C2—O1—C5—O8	107.9 (3)	C6—C7—C17—C29	−176.8 (3)
C2—O1—C5—C4	−7.8 (4)	O8—C7—C17—C18	177.6 (3)
O3—C4—C5—O1	23.9 (4)	C6—C7—C17—C18	59.1 (4)
C6—C4—C5—O1	136.0 (3)	O28—C17—C18—O19	−173.6 (3)
O3—C4—C5—O8	−95.8 (3)	C29—C17—C18—O19	−57.5 (4)
C6—C4—C5—O8	16.3 (3)	C7—C17—C18—O19	63.8 (4)
O3—C4—C6—O9	−168.4 (3)	C17—C18—O19—C20	160.9 (3)
C5—C4—C6—O9	83.3 (3)	C18—O19—C20—O27	−0.8 (6)
O3—C4—C6—C7	76.5 (3)	C18—O19—C20—C21	−180.0 (4)
C5—C4—C6—C7	−31.8 (3)	O27—C20—C21—C22	170.7 (5)
O9—C6—C7—O8	−79.1 (3)	O19—C20—C21—C22	−10.1 (6)
C4—C6—C7—O8	36.6 (3)	O27—C20—C21—C26	−9.3 (7)
O9—C6—C7—C17	41.7 (4)	O19—C20—C21—C26	169.9 (4)
C4—C6—C7—C17	157.5 (3)	C26—C21—C22—C23	2.2 (8)
O1—C5—O8—C7	−107.4 (3)	C20—C21—C22—C23	−177.8 (5)
C4—C5—O8—C7	7.3 (4)	C21—C22—C23—C24	0.6 (10)
C17—C7—O8—C5	−154.4 (3)	C22—C23—C24—C25	−3.5 (9)
C6—C7—O8—C5	−27.9 (3)	C23—C24—C25—C26	3.5 (8)
C4—C6—O9—C10	80.2 (4)	C24—C25—C26—C21	−0.6 (7)
C7—C6—O9—C10	−169.4 (3)	C22—C21—C26—C25	−2.2 (7)
C6—O9—C10—C11	62.9 (4)	C20—C21—C26—C25	177.7 (4)
O9—C10—C11—C12	−113.6 (4)	O28—C17—C29—N30	53.6 (4)
O9—C10—C11—C16	62.6 (4)	C7—C17—C29—N30	175.3 (3)
C16—C11—C12—C13	−1.1 (6)	C18—C17—C29—N30	−62.1 (4)
C10—C11—C12—C13	175.2 (4)	C17—C29—N30—O32	82.8 (4)
C11—C12—C13—C14	−0.1 (6)	C17—C29—N30—O31	−93.9 (4)

### Hydrogen-bond geometry (Å, °)

**Table:**

*D*—H···*A*	*D*—H	H···*A*	*D*···*A*	*D*—H···*A*
O28—H28···O9	0.84 (2)	1.83 (3)	2.628 (4)	157 (5)
C4—H4···O31^i^	1.00	2.44	3.084 (5)	122
C5—H5···O28^ii^	1.00	2.45	3.189 (5)	130
C5—H5···O31^ii^	1.00	2.49	3.352 (5)	144
C18—H18A···O32	0.99	2.46	3.000 (6)	114
C22—H22···O19	0.95	2.41	2.739 (5)	100
C24—H24···O1^iii^	0.95	2.59	3.445 (5)	151
C26—H26···O8^iv^	0.95	2.60	3.514 (5)	162
C29—H29A···O19	0.99	2.57	2.907 (5)	100
C29—H29A···O27^v^	0.99	2.54	3.334 (5)	137
C29—H29B···O8	0.99	2.42	2.824 (5)	104

Symmetry codes: (i) *x*−1, *y*, *z*; (ii) *x*−1/2, −*y*+1/2, −*z*+1; (iii) −*x*+1/2, −*y*, *z*+1/2; (iv) −*x*+1, *y*+1/2, −*z*+3/2; (v) −*x*+1, *y*−1/2, −*z*+3/2.
